# Supplementary material for: Guidance on how to develop complex interventions to improve health and healthcare
Source: BMJ Open. 2019 Aug 15;9(8):e029954. doi: 10.1136/bmjopen-2019-029954 (PMC6701588; doi:10.1136/bmjopen-2019-029954)
Supplement: Supplementary data [file bmjopen-2019-029954supp001.pdf]

Appendix 1 Results of the e-Delphis

The question asked was 'When developing complex interventions to improve health, how important is it to…' with options of very important=5, fairly important=4, somewhat important=3, slightly important=2, not at all important =1. Numbers stating 'Do Not Know' are not reported here.

The column '% agree' presents the percentage ticking very important=5 or fairly important=4.

Consensus was set at 70% agreement for 'very or fairly important' or 'slightly or not important at all'.

The items are presented in order of consensus for developers.

The dark shaded cells are the most frequently ticked options.

No.	Item	Developers							Wider						
		Mode score	% Agree	Not at all important	Slightly important	Somewhat important	Fairly important	Very important	Mode score	% Agree	Not at all important	Slightly important	Somewhat important	Fairly important	Very important
1	Be open to the potential that the final intervention may be different from the initial vision	5	100	0	0	0	0	26	5	100	0	0	0	2	16
2	Report the purpose of the intervention	5	100	0	0	0	0	26	5	100	0	0	0	1	16
3	Report the target population	5	100	0	0	0	1	25	5	100	0	0	0	1	17
4	Clearly define the health problem to be addressed	5	100	0	0	0	1	24	5	100	0	0	0	1	17
5	Review the published evidence before starting to develop an intervention	5	100	0	0	0	1	24	5	100	0	0	0	1	17
6	Be open to failure and going back a step	5	100	0	0	0	2	24	5	100	0	0	0	4	13
7	Ensure team members understand the context in which the intervention will be implemented	5	100	0	0	0	3	23	5	100	0	0	0	3	15
8	Report any use of components from an existing intervention	5	100	0	0	0	4	22	4	100	0	0	0	14	4

9	Report how evidence from different sources informed the intervention development	5	100	0	0	0	5	21	5	100	0	0	0	5	13
10	Report how stakeholders contributed to the intervention development process	5	100	0	0	0	6	20	4	94	0	0	1	14	3
11	Report important uncertainties at the end of the intervention development process.	5	100	0	0	0	7	19	5	78	0	1	3	0	14
12	Look for and take into account evidence that your proposed intervention may not work in the way you intend	4	100	0	0	0	17	9	5	89	0	0	2	2	14
13	Consider the evidence for each substantive intervention component	5	100	0	0	0	11	15	4	94	0	0	1	17	0
14	Have a plan to guide how you will use evidence during the design process	4	100	0	0	0	13	13	4	89	0	0	2	16	0
15	Develop the intervention in an iterative way with regular stakeholder input throughout	5	96	0	1	0	0	25	5	82	0	1	2	3	11
16	Report the context for which the intervention was developed	5	96	0	0	1	0	25	5	100	0	0	0	2	16
17	Ensure the team includes experts in the problem to be addressed by the intervention	5	96	0	1	0	1	24	5	94	0	0	1	1	16
18	Consider facilitators and barriers to future use of the intervention in the real world	5	96	0	1	0	1	24	5	94	0	0	1	1	16
19	Ensure the team specifically includes a behaviour change scientist when the intervention aims to change behaviour.	5	96	0	0	1	2	23	5	83	0	0	3	3	12
20	Document key reasons for decisions made throughout the process	5	96	0	0	1	3	22	5	82	0	0	3	2	12
21	Clearly define the target population: the group of people that will receive the intervention	5	96	0	0	1	3	22	5	100	0	0	0	1	17
22	Ensure high levels of collaboration with stakeholders throughout the development process	5	96	0	1	0	3	22	5	94	0	0	1	2	15

23	Engage all relevant stakeholders	5	96	0	0	1	4	21	5	100	0	0	0	4	13
24	Generate a programme theory/ logic model for how the intervention will have an effect	5	96	0	0	1	4	21	4	94	0	0	1	13	3
25	Identify existing interventions and consider whether they could be adapted	5	96	0	0	1	4	21	5	100	0	0	0	2	15
26	Seek stakeholders' perspectives on several possible versions of the intervention at a very early stage	5	96	0	0	1	4	21	5	89	0	1	1	5	11
27	Carry out feasibility research throughout the intervention development	5	96	0	0	1	5	20	4	89	0	1	1	15	1
28	Consider interactions between parts of the intervention	4	96	0	0	1	20	5	5	94	0	0	1	7	9
29	Develop a plan to integrate patient and public involvement (PPI) into the intervention development process	5	96	1	0	0	5	20	5	83	0	0	3	2	13
30	Stay open minded about the structure, content and delivery of the intervention	5	96	0	0	1	5	20	5	94	0	0	1	6	11
31	Report any changes to interventions required or likely to be required for subgroups	5	96	0	0	1	6	19	4	83	0	1	2	9	6
32	Focus on designing the content, format and delivery of the intervention as much as on gathering or synthesising the evidence to inform it	4	96	0	0	1	17	8	4	78	0	0	4	13	1
33	Have a team large enough to include individuals with all the necessary expertise.	4	96	0	1	0	17	8	4	100	0	0	0	9	9
34	Evaluate important components where there has been team disagreement about aspects of content, format or delivery	4	96	0	0	1	15	10	4	72	0	0	5	13	0
35	Do intervention development quickly	1	96	13	12	0	1	0	2	94	2	15	1	0	0
36	Ensure the team includes members who are skilled at maximising engagement of	5	96	0	0	1	12	13	4	83	0	0	3	11	4

	stakeholders														
37	Involve stakeholders who are members of the target population.	5	92	0	0	2	0	24	5	100	0	0	0	4	14
38	Identify an existing published theory or theories to inform the intervention at the start	4	92	0	1	0	21	3	4	100	0	0	0	17	1
39	Report how any published intervention development approach contributed to the development process	5	92	0	0	2	3	21	4	71	0	0	5	11	1
40	Report how existing published theory informed the intervention development process	5	92	0	0	2	3	21	5	94	0	0	1	5	12
41	Check that the proposed mechanisms of action are supported by early testing	4	92	0	1	1	18	6	5	94	0	0	1	3	14
42	Undertake qualitative data collection to understand the context in which the intervention will be delivered	5	92	0	1	1	8	16	4	83	0	0	3	12	3
43	Consider unintended consequences of the intervention	5	92	0	0	2	9	15	5	94	0	0	1	8	9
44	Ensure all members of the team have the skills and personal qualities to contribute constructively in an interdisciplinary environment	5	92	0	1	1	9	15	4	78	0	1	3	14	0
45	Report any guiding principles, people or factors which were prioritised when making decisions	5	92	0	0	2	11	13	4	83	0	1	2	13	2
46	Collect data from a diverse sample of those who will deliver and receive the intervention	5	92	0	1	1	10	13	4	94	0	0	1	15	2
47	Consider the different levels that the intervention may target and impact (patients, professionals, communities, services)	5	88	0	0	3	2	21	4	94	0	1	0	11	6

48	Draw on a published intervention development approach	4	88	0	2	1	20	3	3	6	0	1	16	1	0
49	Test and refine the programme theory, or logic model, within the development process	5	88	0	0	3	5	18	4	94	0	1	0	14	2
50	Specify gaps and uncertainties in the existing evidence	4	88	0	0	3	16	7	5	94	0	0	1	6	11
51	Ensure the team includes individuals with a strong track record in designing complex interventions	5	88	1	0	2	8	15	4	88	0	0	2	14	2
52	Report how the intervention changed in content and format from the start of the intervention development process	4	88	0	3	0	15	8	4	94	0	1	0	13	4
53	Report the reasons for discarding intervention components that were considered	5	88	0	0	3	9	14	4	88	0	0	2	15	0
54	Use the term 'intervention development' in the title and abstract of any report or publication.	4	85	1	2	1	18	4	3	24	1	2	10	2	2
55	Identify sub-populations that the intervention may need to be adapted for or tailored to	4	85	0	0	4	14	8	4	83	0	1	2	14	1
56	Produce an intervention development protocol detailing the processes to be undertaken to develop the intervention	5	85	0	2	2	10	12	4	88	0	0	2	15	0
57	Apply a published intervention development approach flexibly depending on context	5	84	0	1	3	7	14	4	83	0	0	3	14	1
58	Follow TIDieR guidance when describing the developed intervention	5	80	3	0	2	7	13	5	88	0	0	2	6	9
59	Collect evidence using a diverse range of methods	5	80	0	0	5	9	11	4	100	0	0	0	17	1
60	Draw on more than one existing published theory e.g. both psychological and	4	77	0	2	4	19	1	4	50	1	0	8	9	0

	organisational theories															
61	Have a small sub-team that makes final decisions about the intervention	4	77	1	1	4	19	1	4	61	1	0	6	10	1
62	Use the existing published theories that you have identified to inform the collection of evidence	4	77	1	1	4	16	4	4	67	0	0	6	12	0
63	Agree a process for making decisions within the team about intervention content, format and delivery	5	77	0	0	6	5	15	4	94	0	0	1	15	1
64	Report the intervention development in an open access format (e.g. open access journal, report chapter, website)	4	77	1	1	4	13	7	5	89	0	0	2	5	11
65	Have a funded study with sufficient resources	4	73	0	0	7	14	5	5	88	0	0	2	1	14
66	Establish a set of guiding principles to facilitate decision making about intervention content, format and delivery	4	73	0	1	6	12	7	4	76	0	0	4	11	2
67	Ensure the intervention development team members know their specific roles, rights and responsibilities	5	73	0	0	7	8	11	5	83	1	1	1	2	13
68	Follow every step in a published intervention development approach	2	69	3	15	6	1	1	3	59	5	5	7	0	0
69	Include all stakeholders when making final decisions about the intervention	4	58	0	2	9	12	3	4	83	0	1	2	13	2
70	Ensure the team includes a commissioner or purchaser of health care	2	54	1	13	8	3	1	3	33	1	5	10	1	1
71	Try to design the intervention for use in a wide range of settings	2	52	1	12	7	5	0	3	17	0	1	14	3	0
72	Periodically consider whether additional or alternative existing published theories may be helpful to inform the intervention development.	4	50	1	2	10	13	0	4	67	0	1	5	12	0

73	Have a formal consensus exercise to finalise the content, format and delivery of the intervention	2	50	1	12	7	4	2	3	22	1	3	13	1	0
74	Have equity of decision making amongst key stakeholders and researchers	2	50	2	11	9	2	2	3	22	3	1	10	2	2
75	The team uses methods to enable stakeholders to be creative	3	46	0	1	13	6	6	4	78	0	2	2	12	2
76	Ensure the team includes someone with a background specifically in product or pathway design	4	46	0	3	11	12	0	4	65	0	3	3	11	0
77	Undertake statistical and economic modelling to consider whether an intervention is likely to be worthwhile	2	46	3	9	3	8	3	4	56	0	3	5	9	1
78	Report the background and contribution of those making decisions about the intervention content, format and delivery	3	42	0	4	11	6	5	4	67	1	0	5	10	2
79	Consider the potential cost of several possible versions of the intervention at a very early stage	3	35	0	2	15	8	1	3	0	0	0	18	0	0
80	Have a clear plan of how evidence, data and opinions from different sources will be prioritised and inform the final intervention	3	35	0	2	15	7	2	4	94	0	0	1	16	0
81	Report the time taken to develop the intervention	3	27	1	1	17	4	3	3	17	0	3	14	1	0
82	Consider intellectual property (IP) issues	3	27	3	1	15	4	3	3	39	0	5	6	6	1
83	Report who, when, why and where the original idea for developing the intervention came from	3	27	3	1	15	5	2	4	67	2	1	3	9	3
84	Undertake a quantitative optimisation process to ensure only the strongest components of the intervention are included in the final version	3	27	2	5	15	4	0	3	19	1	2	13	0	0

85	Ensure the team includes someone who has developed a similar intervention	3	23	1	5	17	3	0	3	22	1	3	14	0	0
