# Supplementary material for: Guidance on how to develop complex interventions to improve health and healthcare
Source: BMJ Open. 2019 Aug 15;9(8):e029954. doi: 10.1136/bmjopen-2019-029954 (PMC6701588; doi:10.1136/bmjopen-2019-029954)
Supplement: Supplementary data [file bmjopen-2019-029954supp002.pdf]

Appendix 2 Description of third round e-Delphi participants and consensus group

26 participants completed the developers' e-Delphi third round questionnaire. They were from the UK (n=16), mainland Europe (n=5), Ireland (n=4) and USA (n=1). They had published at least one intervention development study (n=21) or written methodological books or journal articles about intervention development (n=5). Backgrounds are difficult to report simply because people had a range of academic or clinical disciplines which might differ from the title of the department they worked in. We categorised some people under more than one heading. Backgrounds included public health (n=10), applied health research/health services research (n=8), psychology (n=7), nursing (n=6), and allied health professionals (n=1).

18 participants completed the wider stakeholders' e-Delphi third round questionnaire. They were from the UK (n=16), mainland Europe (n=1) and USA (n=1). They were selected for their roles as chairs or members of funding panels (n=5), editors or editorial board members of journals (n=4), commissioners of services (n=3), public and patient involvement (n=3), and other (n=3).

26 participants external to the research team attended the consensus conference in person or by video link/telephone. They were from the UK (n=19), USA (n=3), mainland Europe (n=3), and Ireland (n=1). They were invited in their role as intervention developer (n=13), methodologist (n=4), chair of funding panel (n=3), journal editor (n=3), public and patient representative (n=1), commissioner (n=1), and other (n=1).
